# A Preoperative Algorithm for Loss of Domain Hernia Repair: Stratified Management Using the Tanaka Index in 50 Cases

**DOI:** 10.3389/jaws.2025.14769

**Published:** 2025-09-05

**Authors:** M. Sabari Girieasen, Arunkumar Lakshmanan, Mohamed Farook

**Affiliations:** Institute of General Surgery, Rajiv Gandhi Government General Hospital, Madras Medical College, Chennai, Tamilnadu, India

**Keywords:** hernia repair, TAR, abdominal wall reconstruction, loss of domain, Tanaka Index, Botox, Peritoneal flap

## Abstract

**Background:**

Loss of domain (LOD) in incisional hernias presents a significant challenge in abdominal wall reconstruction. Preoperative preparation of the abdominal wall is crucial to optimize surgical outcomes and prevent abdominal compartment syndrome (ACS). This study aims to develop an algorithm for selecting appropriate patients to undergo preoperative strategies based on the severity of LOD, measured by the Tanaka Index (TI).

**Methods:**

We conducted a prospective study analyzing 50 cases of LOD hernias from a total of 558 incisional hernias treated over a 3-year period (2021–2024). Inclusion criteria were patients aged ≥18 years with a Tanaka Index (TI) > 0.25 who consented to surgery. For cases with TI between 0.25 and 0.30, we performed component separation using the Transversus Abdominis Release (TAR) technique, between 0.31 and 0.35 TAR with peritoneal flap reinforcement. In cases where TI exceeded 0.35, we implemented a structured preoperative preparation protocol involving botulinum toxin (BT) injections and progressive preoperative pneumoperitoneum (PPP) before proceeding with TAR and peritoneal flap reinforcement intraoperatively. In all groups, abdomen was reinforced with a 30 × 30 polypropylene mesh.

**Results:**

The efficacy of these techniques was assessed using both intraoperative and postoperative parameters. Intraoperatively, peak airway pressures (Ppeak and Pplateau) were measured immediately after intubation and after abdominal wall closure. An increase in these pressures was used as an indicator of potential ACS risk. Postoperatively, intra-abdominal pressure was vigilantly monitored using a Foley catheter with serial readings recorded. Among the 50 cases following the algorithm, only two developed elevated intra-abdominal pressures (19 cm H_2_O and 18 cm H_2_O or 14 mmhg and 13.2 mmhg respectively) on postoperative day 0, which normalized by day 3. 6% cases experienced surgical site infections in the immediate postoperative period, and there were no recurrences during a standard 1-year follow-up.

**Conclusion:**

This feasibility study establishes a structured algorithm for managing LOD hernias, tailoring preoperative preparation based on the severity of domain loss rather than standardizing it to all cases. By incorporating intraoperative airway pressure monitoring and postoperative intra-abdominal pressure surveillance, we successfully minimized ACS risk. The proposed approach optimizes fascial closure rates, reduces postoperative morbidity, and demonstrates favorable long-term outcomes.

## Introduction

The number of ventral hernia repairs performed each year worldwide is increasing [1]. The primary reasons behind this are mainly attributed to the rising incidence of obesity [2], and the surge in intra-abdominal surgical procedures [3]. In patients with a history of abdominal sepsis and emergency laparostomies, the ventral defect is frequently left open and later closed using mainly fascial traction or skin grafts. This approach often results in fascial dehiscence or a weak anterior abdominal wall covered only by skin, ultimately leading to the development of large ventral hernias [4]. Complex Incisional Hernias present frequently with large sized defects, combined with a loss-of-domain-situation and in close proximity to bone. It may sometimes present with a full-thickness defect frequently associated with soft-tissue loss, scarring, or infection requiring debridement before reconstruction [5].

Loss of domain (LOD) hernias are large ventral hernias in which simple reduction of herniated contents and primary fascial closure is either not feasible without advanced reconstructive techniques or poses a high risk of complications due to elevated intra-abdominal pressure [4].

The repair of a loss of domain hernia is inherently complex. The abdominal cavity has adapted to the prolonged presence of a substantial portion of its contents outside its confines, within the protruded hernial sac. Reintegrating all the herniated contents into the abdominal cavity in a single surgical procedure can result in a sudden increase in intra-abdominal pressure, predisposing the patient to the potentially fatal complication of abdominal compartment syndrome (ACS). The incidence of ACS in abdominal wall reconstruction is 4.3% [6]. Its Surgical ICU admission and in-hospital mortality was significantly high (58.1%), with an Adjusted Odds Ratio of 3.84 [7, 8]. Hence, to prevent this, it is imperative to acclimatize the abdomen to the anticipated physiological changes through appropriate preoperative and intraoperative techniques.

Preoperative methods involve Intramuscular injection of Botulinum Toxin and Preoperative Progressive Pneumoperitoneum (PPP) [8] where the former induces paralysis of the lateral abdominal wall muscles, enhancing tissue elasticity and promoting the medial displacement of the rectus muscles and the latter subjects the abdomen to a gradual increase in the abdominal pressure with daily insufflation of atmospheric air which passively expands the abdominal cavity, allowing viscera to re-establish right of domain. At the same time, PPP helps to minimize the risks of postoperative abdominal compartment syndrome and the sequelae of fascial closure under tension [9–11].

This study aims to develop an algorithm for preoperative preparation and surgical management of LOD hernias, classifying patients based on the Tanaka Index (TI) [4]. The study hypothesizes that, tailoring preoperative interventions according to the severity of LOD will improve fascial closure rates, reduce ACS risk and render it economically easier on the patient instead of standardising it to all LODs, making this study unique. Furthermore, the study explores how these interventions impact intraoperative abdominal conditions, patient recovery, and long-term outcomes, aiming to establish a standardized approach to complex hernia repair.

## Methods

**FIGURE 1:**
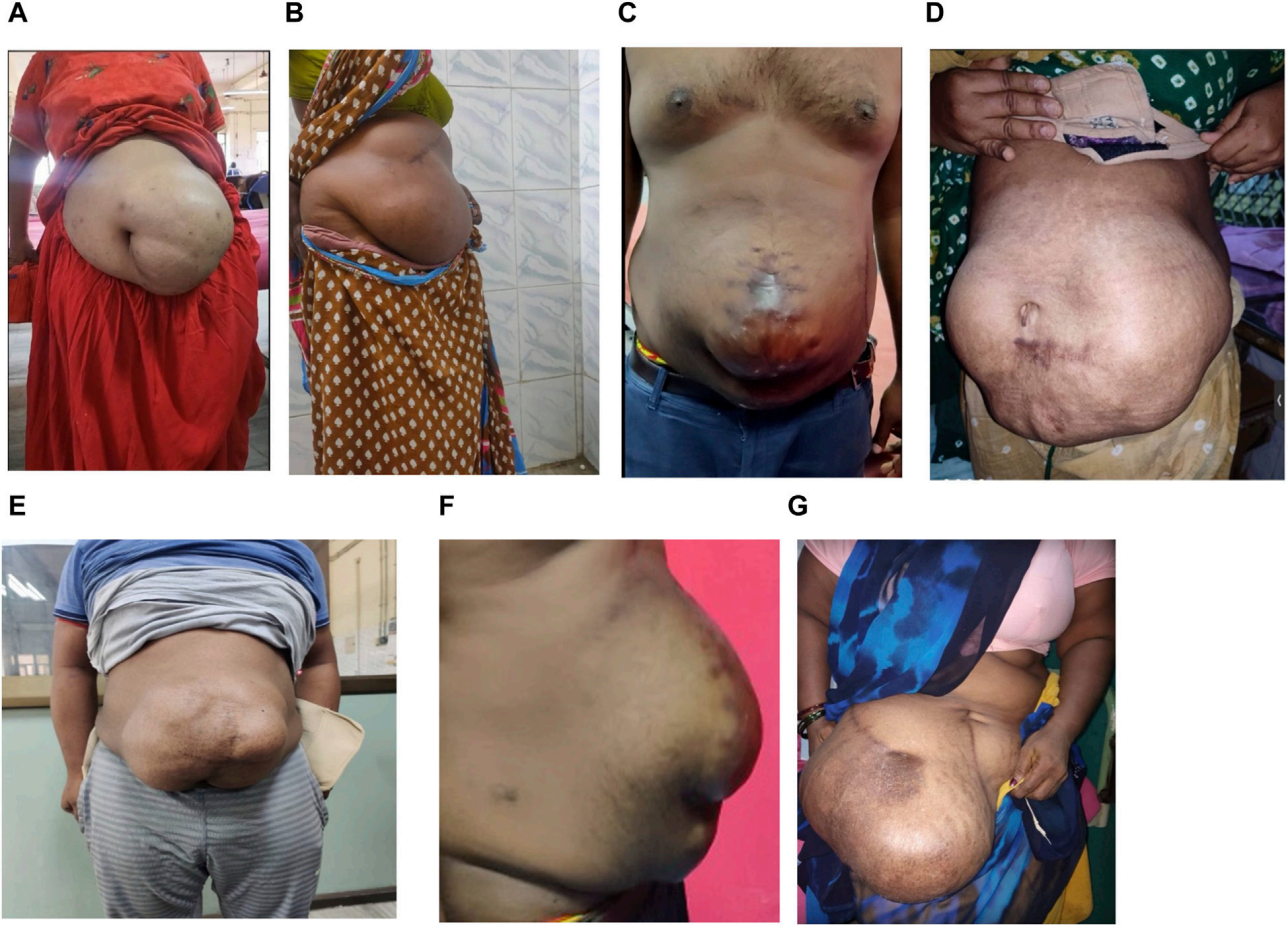
**(A–G)** Clinical picture of LOD cases of varying sizes.

**FIGURE 2:**
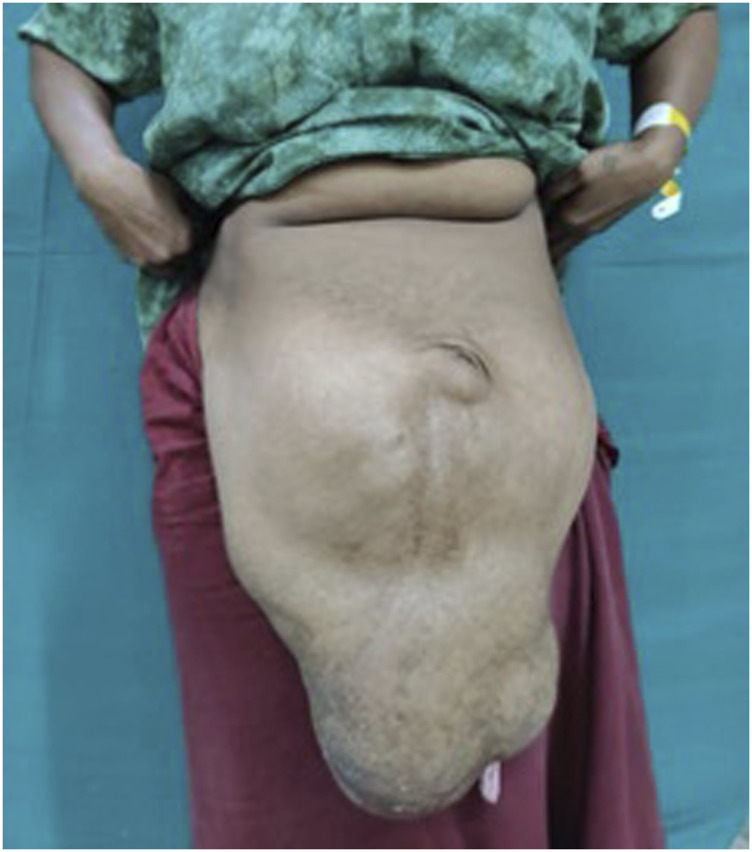
Pre-operative picture of Loss of domain with tanaka index of 0.53.

This prospective study was conducted between 2021 and 2024. Of the 558 patients who underwent surgery for incisional hernias at our hospital, 50 cases of loss of domain (LOD) hernias were selected (Figures 1A–G, 2). Patients were classified primarily based on their Tanaka Index, which is defined as the ratio of hernial sac volume (HSV) to abdominal cavity volume (ACV). A ratio exceeding 0.25 indicates the presence of a loss of domain hernia [4].

Based on this ratio, and using the study by León-Beldarrain et al. (2020) [12], the study developed an algorithm that was approved by our Institutional Ethics Committee. The key distinction between our approach and that of León-Beldarrain et al. (2020) lies in both standardization and threshold selection: while we standardized the technique of posterior component separation (PCS) via transverse abdominis release (TAR) with retrorectus mesh repair for all LOD cases, our threshold for initiating preoperative augmentation (TI > 0.35) was based on institutional experience. At our center, patients with TI between 0.25 and 0.35 had been successfully managed without Botox or PPP, guiding our decision to reserve such interventions for higher-risk cases.

### Algorithm for Incisional Hernias

**FIGURE 3:**
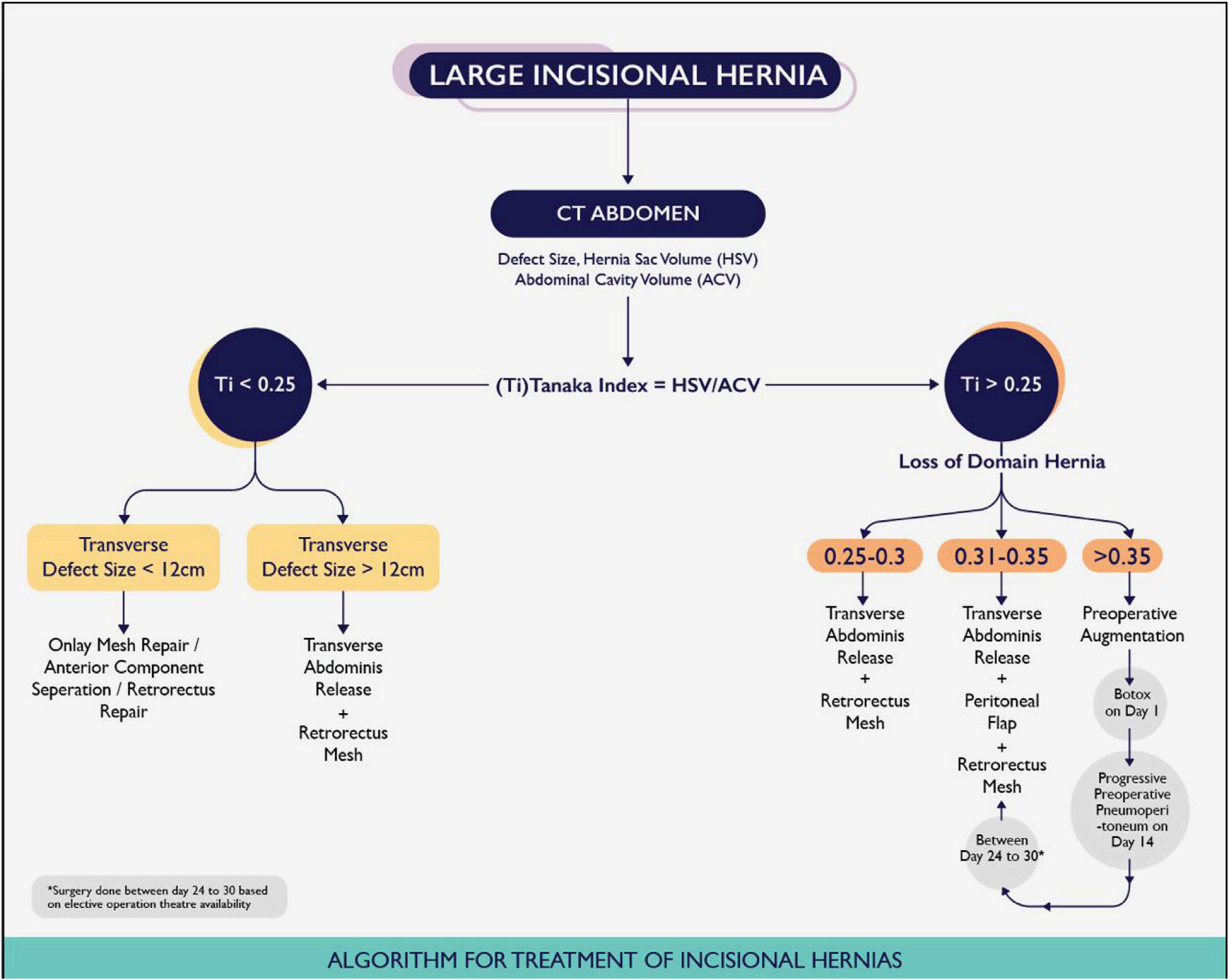
Sabari et al Algorithm for Treatment of Incisional Hernias.

For cases with a Tanaka Index of less than 0.25 (Figure 3), the choice of surgical procedure was determined by the location and size of the hernia. Depending on these factors, anterior component separation, retro-rectus repair, or onlay mesh repair was performed, with transverse abdominis release (TAR) being utilised only in select cases where the defect size exceeded 12 cm. These cases were not included in our study.

For patients with a Tanaka Index between 0.25 and 0.30, posterior component separation via TAR with retrorectus mesh was the preferred surgical approach. When the index ranged from 0.31 to 0.35, the former surgery was combined with an autologous peritoneal flap to facilitate fascial closure. In cases where the index exceeded 0.35, preoperative preparations with botulinum toxin (BT) and progressive preoperative pneumoperitoneum (PPP) were implemented based on reported outcomes and established indications for PPP from previous studies [9, 10, 13]. A pre-anesthetic evaluation was conducted for all patients, including an assessment of lung function. Patients were strictly advised to abstain from smoking and adhere to a weight loss regimen. Additionally, they were actively encouraged to perform incentive spirometry at 2-h intervals everyday. Informed consent was obtained from all patients undergoing the preoperative technique.

Botulinum toxin (BT) injection was administered as an outpatient procedure under ultrasound-guided infiltration 2 weeks prior to the PPP implementation, following the methodology described by Deerenberg EB et al [13]. The anatomical landmarks—costal margin and anterior superior iliac spine—were identified and marked. A line between these two landmarks, half-way between the anterior axillary line and the mid-clavicular line was drawn and divided into three equidistant segments.

**FIGURE 4:**
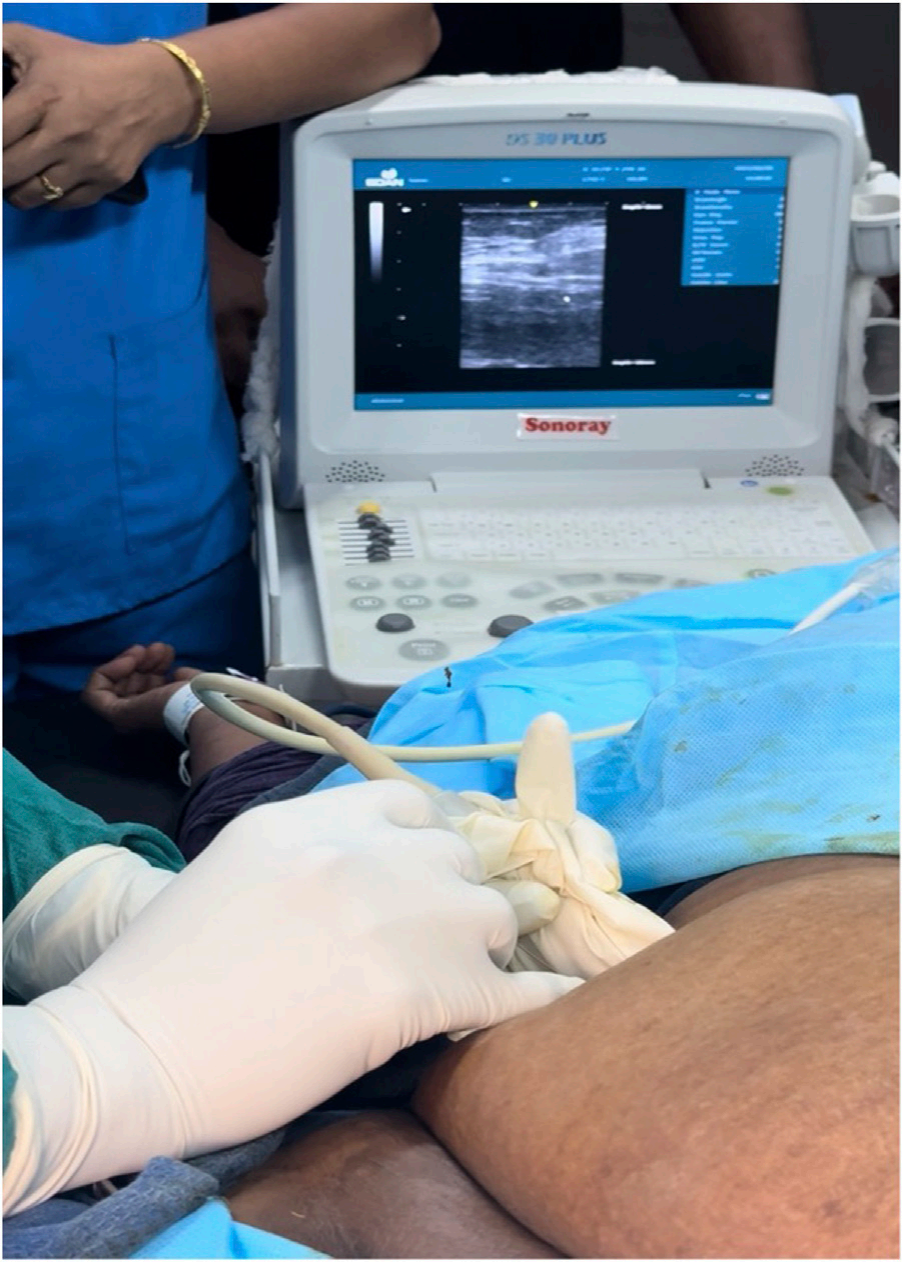
Intraoperative picture of injection of BT into the abdominal musculature, with Ultrasound showing three layers of abdominal muscles in the background.

**FIGURE 5:**
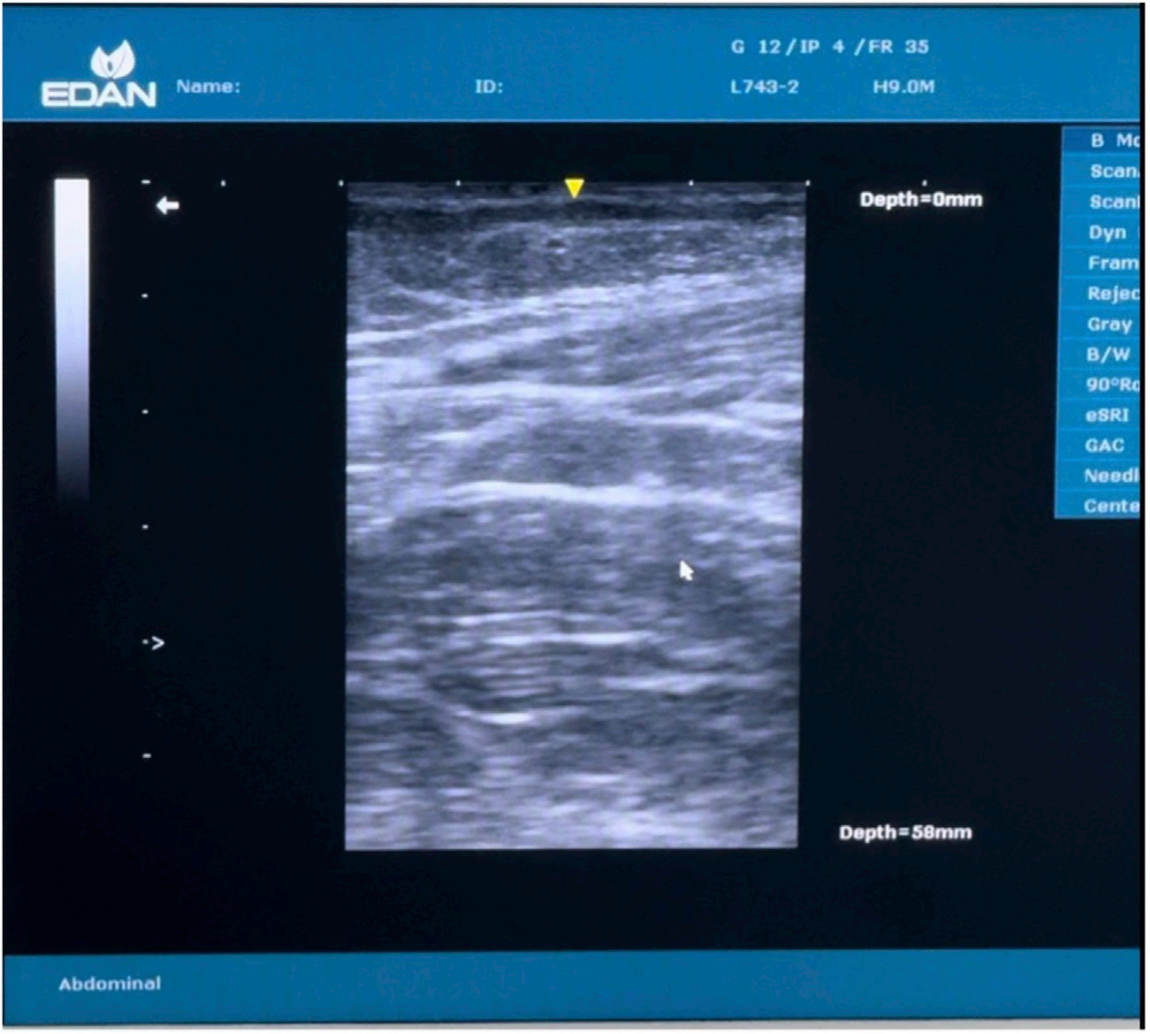
Ultrasound image showing 3 layers.

**FIGURE 6:**
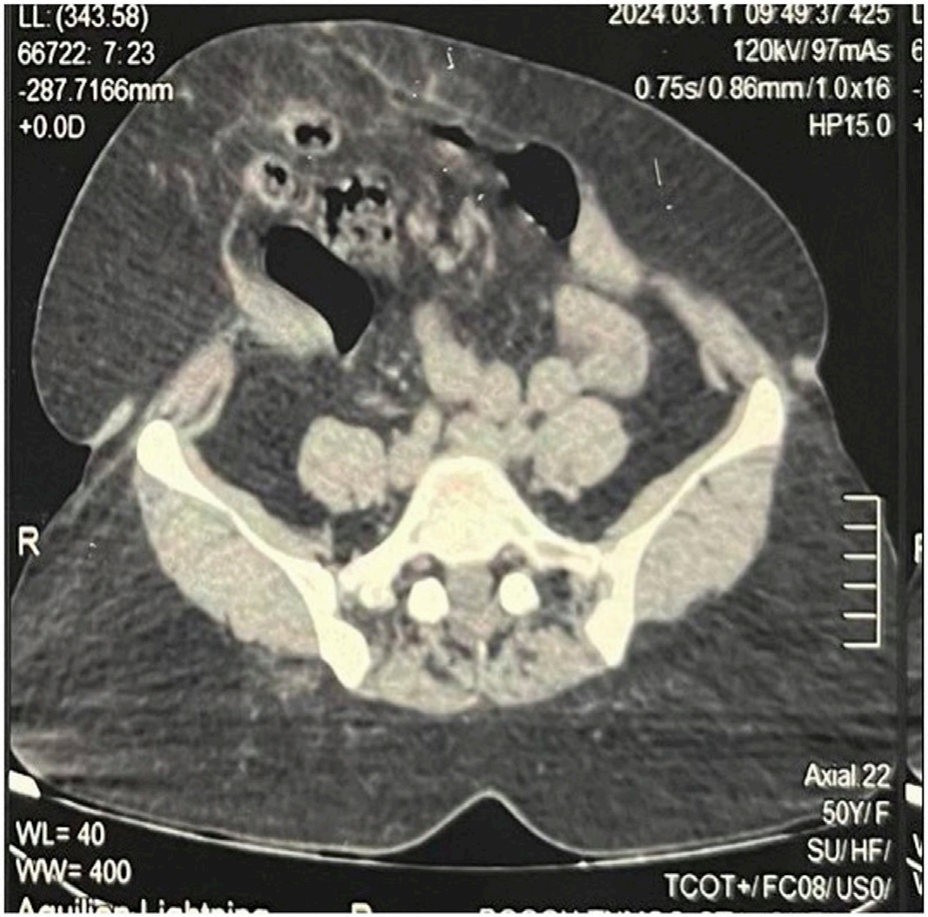
Pre BT CT Abdomen showing tense and shortened abdominal wall muscles.

**FIGURE 7:**
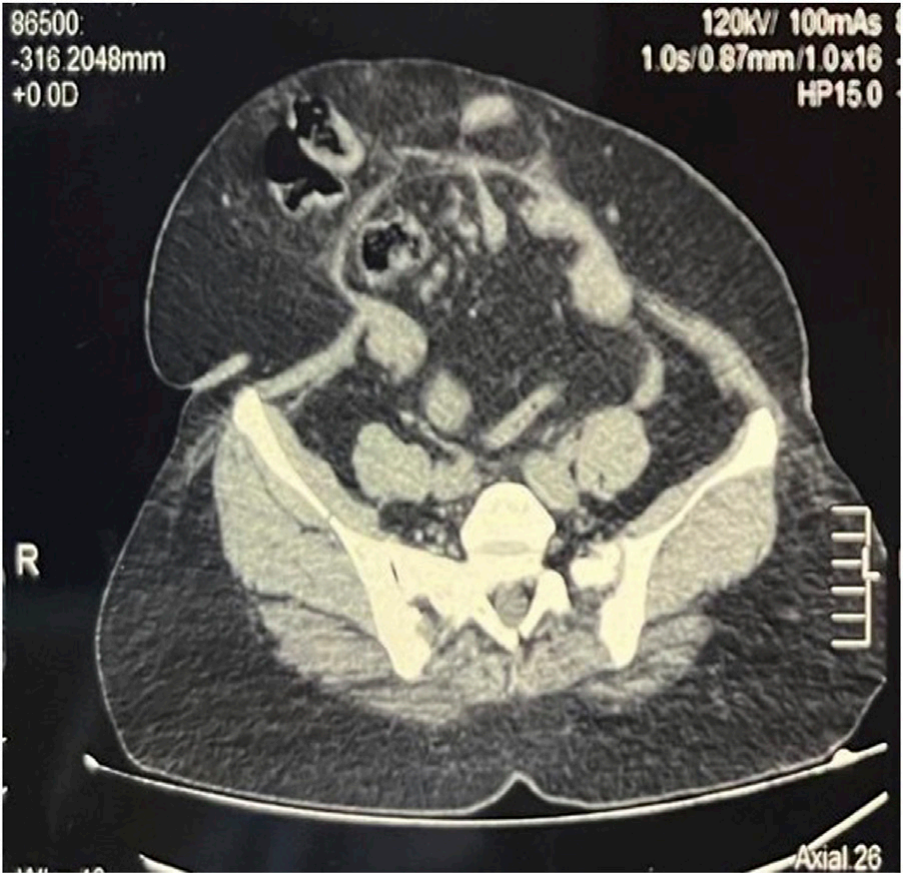
Post BT CT Abdomen showing relaxed and elongated abdominal wall muscles, with rectus moving closer to the midline.

A total of 300 units of botulinum toxin (BT) was reconstituted with 150 mL of normal saline, achieving a concentration of 2 units/mL. Under ultrasound guidance, the flat abdominal muscles were identified, and 25 mL of the solution was injected at each designated site, with approximately 8 mL administered into each muscle group. A total of 150 units was injected on each side of the abdomen. (Figures 4, 5). Pre- and post-Botox CT scans were obtained to assess the increased laxity of the lateral abdominal wall muscles, evidenced by their elongation (Figures 6, 7).

**FIGURE 8:**
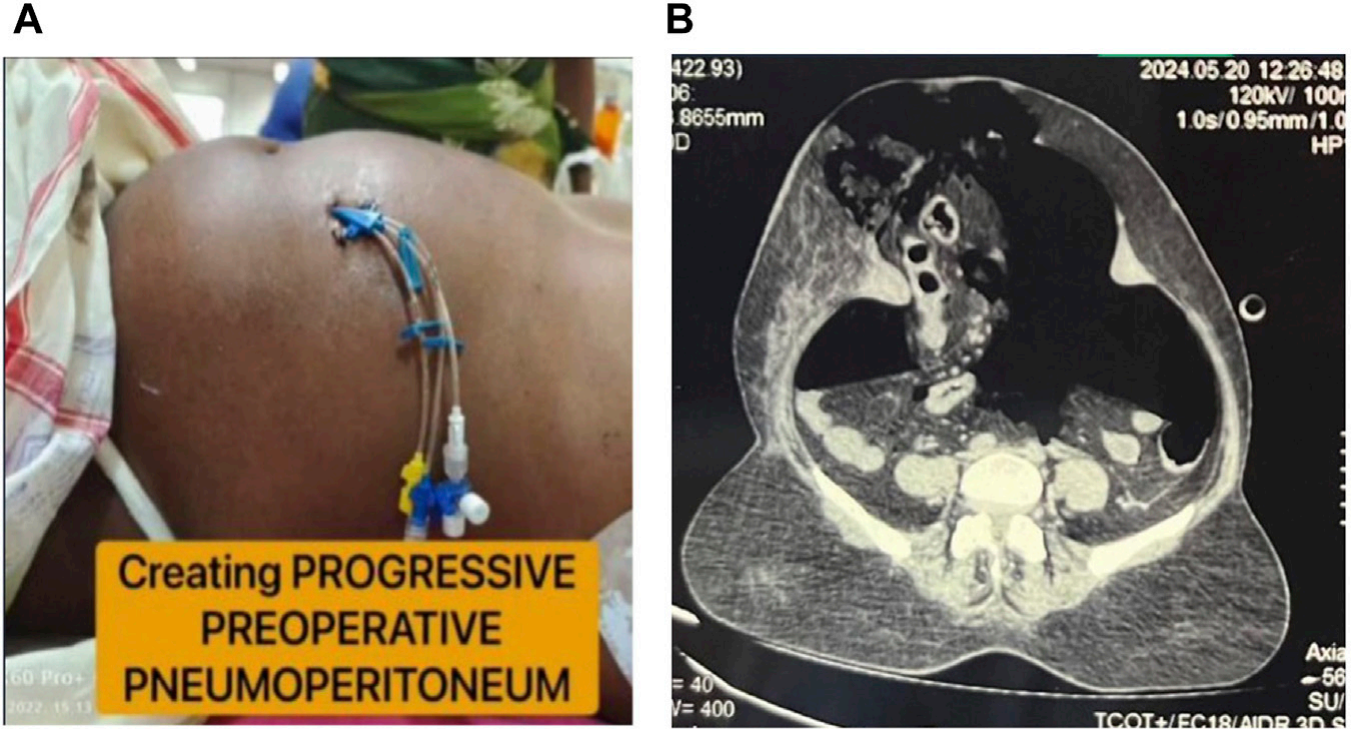
Progressive preoperative pneumoperitoneum. **(A)**: Clinical Picture of PPP with double lumen catheter *insitu*. **(B)**: Post PPP CT showing pneumoperitoneum.

For the implementation of progressive preoperative pneumoperitoneum (PPP), as previously described by [9, 14] an intraperitoneal double-lumen catheter was inserted, preferably in the upper left quadrant, under ultrasound. This procedure was done 14 days after the BT injection when paralyzing effect reaches a maximum [15]. The insertion was performed via an anterolateral approach in an area free from scarring or previous incisions. Initially, a 22G spinal needle was introduced into the peritoneal cavity, ensuring that the tip just crossed the peritoneal space (Figure 8A). A total of 300 mL of unfiltered ambient air was then administered via a syringe, followed by a confirmatory CT scan (Figure 8B). The largest pocket of intraperitoneal air was identified, and an 8Fr pigtail catheter was subsequently placed into that space.

The total insufflation volume was calculated as approximately three times the visceral-intraperitoneal hernia (VIH) volume determined by CT imaging [12]. Progressive insufflation was performed daily or every other day, either in an inpatient or outpatient setting, for a minimum of 7 days, continuing until the day of surgery using a three-way valve. The average volume of insufflation was 800–1,000 mL per day, adjusted according to patient tolerance. Monitoring included assessing abdominal wall distension, discomfort, and dyspnoea, which served as indicators for cessation of insufflation. Complications pertaining to BT injection, such as injection site reactions (pain, swelling, redness or bruising), allergic reactions (itching, dyspnea) or weakened cough, and those to PPP such as shoulder pain, metabolic acidosis, subcutaneous emphysema, pneumothorax, pneumomediastinum, bowel perforation were monitored. A multidisciplinary team including general surgeons, anesthesiologists, and physiotherapists was involved in patient assessment and perioperative care.

Patients underwent surgery under general anaesthesia with epidural cover, and initial peak airway pressures (PAP) including P peak and P plateau were recorded, as a significant increase in PAP before the incision and following fascial closure could potentially lead to alveolar volutrauma [16].

All patients had abdominal wall reconstruction using the posterior component separation technique via transverse abdominis release (TAR), which was carried out unilaterally or bilaterally as per Reinpold et al [17], depending on intraoperative decisions made by the surgeon. Following midline incision, the sac is identified and dissected up to the fascial border of the hernial ring. The sac and peritoneum are then carefully mobilised from the fascial hernial ring, the posterior rectus sheath bilaterally, and the defect’s cephalad and caudal aspects along the linea alba. A longitudinal incision, approximately 0.5 cm medial to the neurovascular bundle, is made along the posterior rectus sheath, ensuring its preservation while allowing for the complete separation of the rectus abdominis muscle from the posterior rectus sheath up to the lateral edge of the rectus compartment. The retromuscular plane is subsequently developed towards the junction of the posterior and anterior rectus sheaths, creating a suitable space for further surgical reconstruction. Released Transversus Abdominis Muscle is pushed away to enter the space between the Transversus Abdominis muscle and Transversalis fascia. Inferiorly, the space of Retzius is entered and Cooper’s ligament visualized. Superiorly, the retro muscular plane can be extended cephalad to the xiphoid and diaphragm if needed.

**FIGURE 9:**
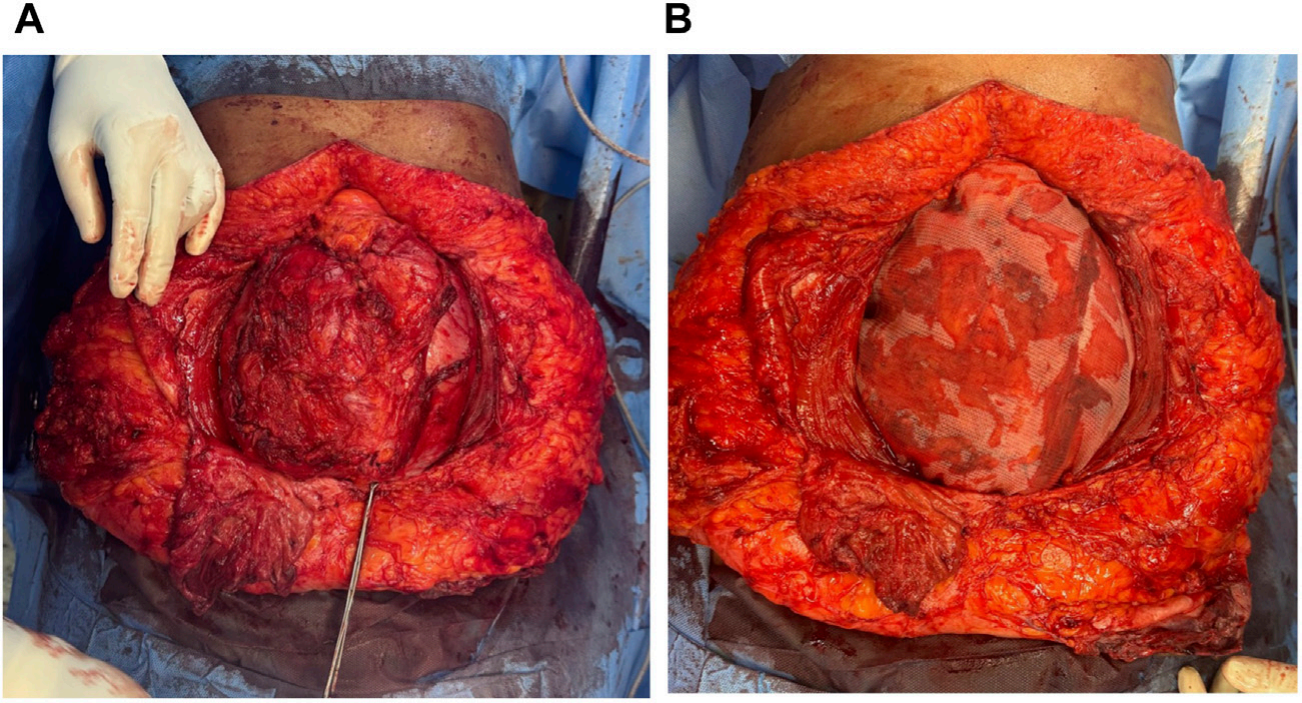
Intraoperative pictures. **(A)**: Transverse Abdominis Release with peritoneal flap. **(B)**: 30 × 30 cm polypropylene mesh placed in Retro rectus plane.

For patients with a Tanaka Index exceeding 0.3, the “Swinging Door” technique, a peritoneal flap method, was employed alongside transverse abdominis release (TAR) [18, 19] (Figure 9A). This technique utilises excess tissue from the hernial sac to close the fascial defect, effectively enlarging the abdominal domain at the herniation site without weakening the lateral abdominal wall [20].

A 30 × 30 cm polypropylene mesh was placed in the underlay space to cover the defect, extending laterally into the retroperitoneum and secured with 2-0 Prolene sutures (Figure 9B). A Romovac negative suction drain tube was positioned over the mesh within the retrorectus plane to facilitate drainage. Finally, the anterior rectus sheath was meticulously closed to restore the linea alba.

Redundant skin and subcutaneous tissue were excised, and a second subcutaneous drain tube (DT) was inserted. The skin was then sutured without tension, and a tight compressive dressing was applied. An increase in Peak Airway Pressures was noted. A rise of >10 cm H_2_O suggested imminent abdominal compartment syndrome [21]. As described by Novitsky in his book *Hernia Surgery: Current Principles*, the study standardized an increase of >8 cm H_2_O (>6 mm Hg) as significant and continued patients on elective ventilation [22]. We vigilantly monitored intra-abdominal pressure for Abdominal Compartment Syndrome (ACS) using a Foley catheter and a central venous pressure (CVP) manometer, following the previously described technique in Rao P et al and Hunter JD et al [23, 24]. Serial readings of IAP were noted to identify early signs of ACS. Post Extubation, patients were advised to perform incentive spirometry and early ambulation. Epidural top-ups were given with tramadol till day 3. The DTs were removed on Post operative day 3 or if output was <25 mL/day, whichever occured later. Sutures were removed between Post operative days 10–14 in healthy wounds.

We considered various parameters, including age, sex, duration of hernia, past surgical history, smoking history, chronic medical conditions, and CT findings such as defect size and Tanaka Index. Additional factors assessed included intraoperative procedures, elevation in peak airway pressures, length of hospital stay, postoperative pain, postoperative complications (both local wound and systemic), date of discharge, postoperative follow-up, and recurrence rate.

## Statistical Analysis

Data analysis was performed using IBM SPSS Statistics version 21.0. The Kolmogorov-Smirnov test was conducted to assess data normality, yielding a non-significant p-value, indicating that the data were normally distributed. Due to the small cohort size, non-parametric test was applied to check the difference between groups, descriptive statistics were reported as median and interquartile range (IQR) for continuous variables, while categorical variables were presented as frequencies and percentages.

Comparisons between three independent groups were conducted using the Kruskal-Wallis test for continuous variables. The Chi-square test or Fisher’s exact test was applied to examine associations between categorical variables, as appropriate.

A p-value <0.05 was considered statistically significant.

## Results

Normality of continuous variables was assessed using the Kolmogorov–Smirnov test. A p-value greater than 0.05 indicates that the variable follows a normal distribution. In this study, Age (p = 0.06), BMI (p = 0.20), Tanaka Index (p = 0.05), Defect Size (p = 0.164), and all intra-abdominal pressure values from post-operative days 0–3 (IAPONPOD0 to IAPONPOD3, all p = 0.20) showed no significant deviation from normality. However, Duration of Hernia had a p-value of 0.001, indicating a statistically significant deviation from normality (Table 1). Although most variables satisfy the assumptions for parametric testing, non-parametric methods will be applied due to the small sample size of the cohort, to ensure more reliable and conservative results.

**TABLE 1:** Normality test results for continuous variables (PAP normality).

Tests of normality	
Variables	Kolmogorov-smirnova
	Sig
Age	0.06
BMI	0.20
Tanaka index	0.05
Defect Size	0.164
Duration of Hernia	0.001
IAPONPOD0	0.20
IAPONPOD1	0.20
IAPONPOD2	0.09
IAPONPOD3	0.20

The Q-Q plots for the normally distributed variables (Age, BMI, Tanaka Index, Defect Size, and IAP ON POD0 to POD3) revealed points closely aligned with the diagonal reference line, supporting the normality suggested by statistical tests. The Q-Q plot for Duration of Hernia showed a clear deviation, confirming its non-normality. Despite overall normality in the visual and statistical tests, non-parametric tests will be used for analysis in light of the small cohort size, to minimize the risk of assumption violations and enhance the robustness of the findings.

### Demographic Characteristics

A total of 50 patients with loss of domain (LOD) hernias were included in this study. Groups (I,II,III) were categorized based on Tanka Index (Table 2). The median age of the cohort was 52 years (range: 36–66 years), with a male-to-female ratio of 1:1.78 (Table 3). The median BMI and the duration of hernia amongst the patients was at an increasing trend across the 3 groups and the association was found to be statistically significant (p value < 0.001). Age, sex distribution, ASA classification, and number of prior surgeries did not differ significantly between groups. 28% (n = 14) were active smokers who were about 2.5 times less than nonsmokers (n = 36). Patients with hernia following just a single abdominal surgery was highest in Group I (n = 16), whilst Group III had the highest incidence of hernia following 3 surgeries (n = 4). Among the patients, all of whom have had a previous history of abdominal surgery, 44% (n = 22) had undergone an emergency laparotomy. 68% (n = 22) of females had history of Obstetric procedures. Preoperative comorbidities included hypertension (32%), diabetes mellitus (28%), and chronic obstructive pulmonary disease (12%). However, number of patients with preoperative comorbidities was more in Groups II and III.

**TABLE 2:** Categorisation of groups.

Management group	Tanaka index
Group 1	0.25–0.30
Group 2	0.31–0.35
Group 3	>0.35

**TABLE 3:** Baseline characteristics of patients (n = 50) after separating by management groups.

Variables		Group I (n = 24) TAR	Group II (n = 14) TAR + peritoneal flap	Group III (n = 12) TAR + peritoneal flap + Botox	p value
		Med (IQR)	Med (IQR)	Med (IQR)	
Age (in years)		50 (48,54.5)	49 (48,52)	53 (48,56)	0.681
BMI (kg/m^2^)		24.7 (23.85,26.45)	29.1 (28.3,29.8)	33.55 (32.6,36.6)	<0.001
Sex		n (%)	n (%)	n (%)	0.838
Male		10 (41.7)	4 (28.6)	4 (33.3)	
Female		14 (58.3)	10 (71.4)	8 (66.7)	
Duration of Hernia (in years) Median (IQR)		1.5 (1,2.5)	4 (2,5)	7 (6,8)	<0.001
ASA Classification		n (%)	n (%)	n (%)	0.235
1		10 (41.7)	2 (14.3)	2 (16.7)	
2		14 (58.3)	10 (71.4)	6 (50)	
3		0	2 (14.3)	4 (33.3)	
Risk Factors		n (%)	n (%)	n (%)	
Smoking		6 (25)	4 (28.6)	4 (33.3)	0.919
No. of abdominal surgeries	1	16 (66.7)	8 (57.14)	6 (50)	0.724
	2	6 (25)	4 (28.57)	2 (16.67)	
	3	2 (8.3)	2 (14.28)	4 (33.33)	
Comorbidities		n (%)	n (%)	n (%)	
DM		8 (33.33)	4 (28.57)	2 (16.67)	0.655
HTN		10 (41.67)	4 (28.57)	2 (16.67)	0.358
COPD		0 (0)	4 (28.57)	2 (16.67)	-
Past Surgical History		n (%)	n (%)	n (%)	0.670
Emergency Laparotomy		10 (41.7)	6 (42.9)	6 (50)	
Puerperal Sterilisation		8 (33.3)	2 (14.3)	2 (16.7)	
Cesarean section		2 (8.3)	6 (42.9)	2 (16.7)	
Previous Hernia Surgery		2 (8.3)	-	2 (16.7)	
Colectomy		2 (8.3)	-	-	

### Preoperative and Intraoperative Characteristics

As assessed on CT imaging, the median transverse defect size was increasing across the 3 groups. Among patients who received preoperative progressive pneumoperitoneum (PPP), the median air insufflation volume was 13 L.

All patients underwent posterior component separation with transverse abdominis release (TAR), with 26 patients (52%) requiring additional peritoneal flap augmentation. However only 12 of these 26 patients needed preoperative augmentation (Table 4). Intraoperative peak airway pressure (PAP) monitoring revealed significant difference (p value – 0.002), a median increase of 8.2 cm H_2_O for Group II at the time of closure which was higher than other Groups (Table 4). The blood loss increased over the 3 groups and was statistically significant (p < 0.001). A significant rise in PAP (>8 cm H_2_O) was noted in 26 patients (52%), all of whom required elective postoperative ventilation.

**TABLE 4:** Pre and intra-operative characteristics.

Variables	Group I (n = 24)	Group II (n = 14)	Group III (n = 12)	p value
	Med (IQR)	Med (IQR)	Med (IQR)	
Transverse defect size (in cm)	13.4 (12.5–14.3)	15.6 (15.0–16.5)	17.7 (17.0–18.5)	0.056
Total Volume of air insufflated in PPP (in litres) (n = 12)	n.a		13 (9.4,14)	
Increase in Peak Airway Pressure (cm H_2_O)	6.8 (3.4,8.2)	8.2 (8.2,8.2)	4 (2.7,5.4)	0.002
Blood loss (in mL)	180 (155,205)	240 (220,250)	275 (270,300)	<0.001
Primary Surgeon A/B/C	Surgeon (n)	Surgeon (n)	Surgeon (n)	
	A (11) B (6) C (7)	A (14)	A (12)	
Assisting Surgeon B/C	B (12) C (12)	B (8) C (6)	B (4) C (8)	

### Postoperative Outcomes

**FIGURE 10:**
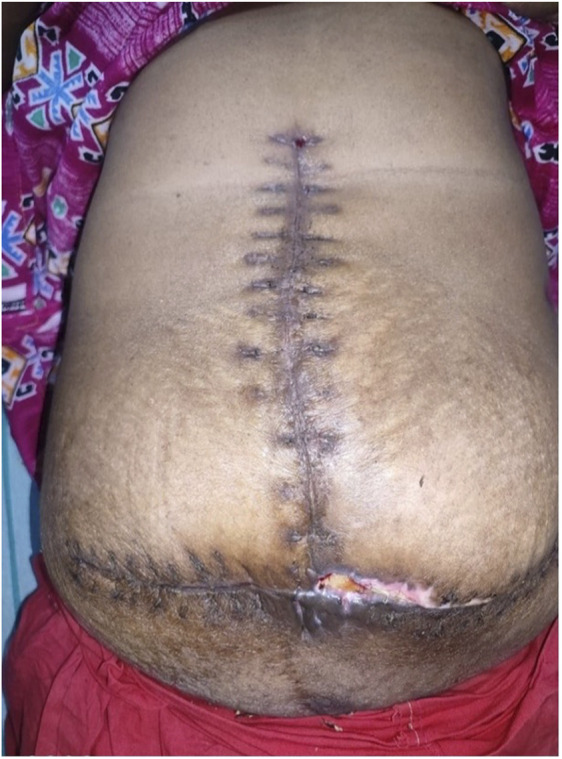
Postoperative picture with wound dehiscence.

All patients were monitored from the postoperative day (POD) 0 for intra-abdominal pressure using a central venous pressure (CVP) manometer, which remained comparable across groups until POD 3 (all p > 0.2), with no values exceeding thresholds associated with abdominal compartment syndrome (ACS), suggesting the protocol was effective in mitigating this risk (Table 5). Patients who required elective postoperative ventilation (n = 26) were successfully weaned off within 48 h. However, the need for postoperative ventilator support was significantly higher in Group 2 (85.7%) compared to Group 1 (50%) and Group 3 (16.7%) (p = 0.002). With respect to complication, superficial surgical site infections (SSI) were seen only in groups 2 and 3 (14-17%) (Figure 10). Seromas were significantly more common in Group 3 (33.3%) versus Group 2 (14.3%) and absent in Group 1 (p = 0.014). Both these complications were managed conservatively. Systemic complications included acute respiratory distress syndrome, defined by acute onset, bilateral lung infiltrates on chest radiography or CT scan of a non-cardiac origin, and a PaO_2_/FiO_2_ ratio of less than 300 mm Hg with the requirement of positive end-expiratory pressure (PEEP) or continuous positive airway pressure (CPAP) of greater than or equal to 5 cm H_2_0, which occured in 8% of cases [25]. The other complication was transient acute kidney injury, defined as temporary decline in kidney function with a return to baseline values in 48 h, was noted in 4% of cases [26]. Both the complications occurred in Group II. The median length of hospital stay for group II was 12 days which was the highest among the groups. Overall, while defect size and disease severity increased with the Tanaka Index, the structured intraoperative and postoperative protocols effectively prevented ACS, although systemic complications and ventilator dependence were more frequent in intermediate-risk patients.

**TABLE 5:** Post-operative characteristics.

Variables		Group I (n = 24)	Group II (n = 14)	Group III (n = 12)	p value
		Med (IQR)	Med (IQR)	Med (IQR)	
	POD-0	13.25 (12,14)	16 (13.5,17)	14.25 (11,16)	0.250
Intra-abdominal pressure (cm H_2_O)	POD-1	12.5 (11.5,13.75)	13 (11.5,15.5)	12.5 (10.5,14)	0.491
	POD-2	12 (11,12)	11.5 (11,13)	11.75 (10,13.5)	0.803
	POD-3	10.5 (10,11.25)	11 (10,12)	10.25 (9.50,11)	0.476
Length of Stay (in days)		11 (9, 12)	12 (10, 14)	9.5 (8, 12)	0.524
		n (%)	n (%)	n (%)	
Patients under postop ventilator		12 (50)	12 (85.71)	2 (16.67)	0.002
Day of Extubation (n = 26)		n (%)	n (%)	n (%)	
POD 1		8 (33.33)	8 (57.14)	-	0.008
POD 2		4 (16.67)	4 (28.57)	2 (16.67)	
Postop Complications		n (%)	n (%)	n (%)	
SSI		-	2 (14.29)	2 (16.67)	0.131
Seroma		-	2 (14.29)	4 (33.33)	0.014
Systemic Complications		-	6 (42.86) 4 AKI/2ARDS	-	0.002

### Follow-Up and Recurrence

At a median follow-up of 12 months, 100% of patients achieved successful fascial closure without recurrence. One patient experienced recurrence during his 2-year follow-up who had a Tanaka Index of 0.34 at baseline. None of the patients required reoperation within the follow-up period. This study demonstrates that posterior component separation with TAR, with or without peritoneal flap augmentation, is an effective technique for LOD hernias. While preoperative interventions remain valuable, our algorithm for surgical planning based on defect size and intraoperative PAP monitoring proved to be a safe and effective alternative in resource-limited settings.

## Discussion

A critical concern when forgoing preoperative preparation in LOD patients is the potential risk of abdominal compartment syndrome (ACS) due to a tense fascial closure during surgery. To prevent this preoperative augmentation was implemented in LOD hernias. However, this proved to be costly for patients and also required prolonged duration of preoperative stay.

When compared with previous studies, our findings align with those of León-Beldarrain et al. (2020), who advocated for the routine use of PPP and BT in all cases of LOD hernias. That said, our study sought to explore whether TAR along with a retrorectus mesh could be a viable alternative for cases with a Tanaka Index between 0.25 and 0.30, particularly in resource-limited settings. Our results indicate that TAR, with or without a peritoneal flap, facilitated fascial closure without excessive lateral wall tension, offering a practical solution for situations where preoperative techniques are unavailable. But this could also create tense closure leading to Intra abdominal Hypertension. To prevent this, monitoring of Abdominal Compartment Syndrome was performed. Intraoperative peak airway pressure (PAP) as a surrogate marker for intra-abdominal hypertension [27] was monitored. A significant rise in PAP at the time of closure suggests the development of varying degrees of intra-abdominal hypertension, which can have severe postoperative respiratory consequences. This is primarily due to increased chest wall elasticity, elevated intrathoracic pressure, and subsequent reductions in lung volume, resulting in atelectasis [28]. However, increasing the natural compliance of the abdominal wall through positive ventilatory support allows for adaptation to elevated intra-abdominal pressure [29]. For this reason, in our study, patients demonstrating a marked intraoperative rise in PAP were electively ventilated postoperatively, to ensure a gradual adaptation to the rising abdominal pressure. Their Intra-Abdominal Pressures (IAP) were vigilantly monitored by Foley’s catheter once every 4 h during the first post-operative day.

This study evaluates the effectiveness of the posterior component separation technique such as Transverse Abdominis Release (TAR) with retrorectus mesh placement in conjunction with a peritoneal flap for loss of domain (LOD) hernias, particularly in cases with a Tanaka Index greater than 0.3. Preoperative techniques such as progressive preoperative pneumoperitoneum (PPP) and botulinum toxin (BT) injections remain highly successful for managing LOD hernias even in cases with a Tanaka Index exceeding 0.25 [12]. But, their widespread application in government hospitals in India presents significant challenges. BT is difficult to procure in such settings, and PPP necessitates prolonged inpatient admission (often up to 10 days preoperatively), increasing hospitalization costs, which is particularly burdensome for patients of low socioeconomic status who seek treatment in these centers. Given these limitations, the study aimed to develop an alternative algorithm tailored to such constraints, employing TAR for smaller-grade LOD hernias while reserving preoperative augmentation for more extensive defects.

Nonetheless, this does not suggest that TAR is superior to surgeries done with preoperative augmentation. Rather, it underscores the need for adaptable surgical strategies in hospitals where access to BT and prolonged preoperative admissions for PPP is constrained.

Despite the promising findings, our study has certain limitations. The use of elective postoperative ventilation delays early recovery, can cause Ventilator Associated Pneumonia (VAP), Ventilator Induced Lung Injury (VILI) and may subject patients to additional physiological stress [30, 31]. Furthermore, vigilant monitoring for abdominal compartment syndrome in the early postoperative period remains challenging due to limited medical personnel and infrastructure constraints in resource-limited settings.

Further studies should also be conducted to examine whether alternative preoperative techniques such as tailored PPP protocols with shorter hospital stays could be developed to accommodate financial and logistical constraints in government hospitals. Future efforts should be applied to increase the threshold of elective post operative ventilation and hence subject lesser patients to VAP, VILI and stress.

### Conclusion

The value of a systematic strategy to the management of loss of domain (LOD) hernias has been demonstrated by this study. Customised preoperative and intraoperative techniques after classifying patients according to the Tanaka Index, greatly increased fascial closure rates and has shown no complications of abdominal compartment syndrome. Botulinum toxin injections and progressive preoperative pneumoperitoneum (PPP) enhanced abdominal compliance for very large hernias, enabling safer hernia repair.

For intra-abdominal hypertension, intraoperative airway pressure monitoring has been shown to be a trustworthy surrogate marker, enabling early intervention such as elective postoperative intubation, to avoid problems after surgery. Additionally, postoperative intra-abdominal pressure surveillance through Foley catheter measurements was critical in reducing morbidity.

Our findings show that a targeted, patient-specific strategy is both economical and successful. The long-term success of this approach is further supported by the lack of recurrence during a 1-year follow-up period. Future research should focus on validating these findings through larger multi-center studies and exploring additional cost-effective alternatives for preoperative preparation.

## Data Availability

The raw data supporting the conclusions of this article will be made available by the authors, without undue reservation.
